# Evaluation of selected elements of fitness and physiological diagnostics of military pilots as a factor in flights safety

**DOI:** 10.3389/fphys.2025.1558786

**Published:** 2025-05-02

**Authors:** Zbigniew Wochyński

**Affiliations:** Department of Air Transport Safety, Polish Air Force University, Deblin, Poland

**Keywords:** aviation-synthetic efficiency test (ASET), L-1 manoeuvre, psychomotor performance, lipid index, biochemical indexes, aviation medicine, flight safety, physical capability

## Abstract

The author of the publication drew attention to factors that threaten the safe performance of a military aviation mission. The aim of the article is to present an effective method for diagnosing psychomotor skills, monitoring physical fitness and selecting pilots of highly maneuverable aircraft, as well as selected exercise markers of high diagnostic value for assessing the adaptation (efficiency) of the pilots’ body. First, the focus was on the practical direction of preventing aviation accidents. Based on many years of research on cadet pilots, the author developed the Aviation-Synthetic Efficiency Test (ASET), which aims to select candidates for aviation and assess the performance of an anti-overload maneuver involving appropriate skeletal muscles. The test was used to monitor the training process in order to obtain an optimized physical load on the body. The factor strengthening the implementation of an effective pilot training process was the assessment of the behavior of the WS lipid index as confirmation of the training effects in the G-strain centrifuge test. The author attributes special diagnostic value to lipid indices for pilots of highly maneuverable aircraft. A diagnostic and training device was developed and used to monitor the special training process in terms of assessing the psychomotor level of pilots in extreme conditions of the pilot’s work environment. Studies were also conducted in the special training process on special aviation gymnastics devices (SAGI). Physiological and biochemical indicators were used in these studies, emphasizing the high diagnostic value in relation to the physical fitness and health of pilots. The aspects of the research presented in this article contribute to the pilots’ compliance with the balance between the performance of tasks and their physical capabilities, which undoubtedly increases flight safety.

## Introduction

The physical fitness of a military pilot is one of the most important factors in flight safety. Among multiple endurance tests, the fitness tests for targeted and special flight preparation are particularly noteworthy. The new equipment possessed by the Polish Air Force (e.g.,: F-16 aircraft), as well as those that will be used for military aviation in the near future, require appropriate (adjusted) fitness and physical capability. Over the years, it has been noted that more modern equipment entails higher fitness, performance and psychomotor requirements for pilots. This is caused by the fact that the pilot’s body is more exposed to the negative factors of flight, which include: overload, airsickness, disorders of spatial orientation (balance) and eye-hand coordination under acceleration + Gz (head-leg direction) as well as - Gz (leg-head direction). Often these factors caused aviation accidents. Their analysis led to the conclusion that adequate psycho-physical fitness of the pilot would be the counteracting factor in a majority of these mishaps. Between 1982 and 2001, human factor was found to be the cause of accidents in airworthy highly maneuverable aircraft (Lyons et al., 2004). When the probable causes of accidents were investigated, it became apparent that some pilots had been insufficiently prepared to counteract the negative flight factors of accelerations occurring under extreme conditions of their job (Lyons et al., 2004). Changes in the execution of physical training programmes (Wochyński et al., 2010) and fitness diagnostics introduced for pilots (Klukowski et al., 1992) have proved this view. The adopted exercises of skeletal muscle groups involved in the performance of the anti-G straining manoeuvre (Chen et al., 2004; Oksa et al., 1996; Wojtkowiak et al., 2006) led to a significant reduction in air accidents. For this purpose, measures have been taken to monitor the fitness level of pilots in the training process with a particular focus on maintaining the level of the anti-overload manoeuvre. Also, biochemical indexes have been used to assess exercise adaptation in the extreme conditions of the pilot’s working environment. Preparing pilots for the extreme conditions of their work requires a high degree of involvement of specialists in aviation medicine, physical culture and psychology, in order to ensure a safe execution of a combat mission. A practical analysis of the physical fitness of pilots demonstrated that a test was necessary for pilots to comprehensively diagnose the level of motor abilities. On this basis, it would be possible to initiate the process of implementing targeted physical preparation and check its effects. Next, it is essential to begin special preparation on the Special Aviation Gymnastic Instruments (SAGI). SAGI are an important element of pilot training in the field of eye-hand coordination, spatial orientation, balance and airsickness. These include devices such as: looping, gyroscope and Rhine wheel. The SAGI training process heavily burdens the muscular, circulatory, nervous and hormonal systems. The exercises were performed with low intensity, but caused physiological changes similar to those in competitive athletes (Wochyński and Sobiech, 2015). These changes occurred due to the specificity of the exercises, which are classified as extreme due to the occurring accelerations + Gz (head-legs direction) and - Gz (legs-head direction). The training process required the use of exercise markers in order to monitor the proper achievement of exercise adaptation.

The aim of this article is to present, on the basis of many years of experience, a method for diagnosing psychomotor abilities, monitoring physical fitness and selecting highly maneuverable aircraft pilots as well as the use of selected exercise markers of high diagnostic value to determine the state of achieved adaptation (capability) of the pilots’ bodies, thus influencing flight safety. The author of the article asked whether selected elements of the fitness and physiological diagnostics of military pilots may be of significant importance in the context of flight safety due to the human factor?

## Monitoring physical fitness with the aviation-synthetic efficiency test

**FIGURE 1:**
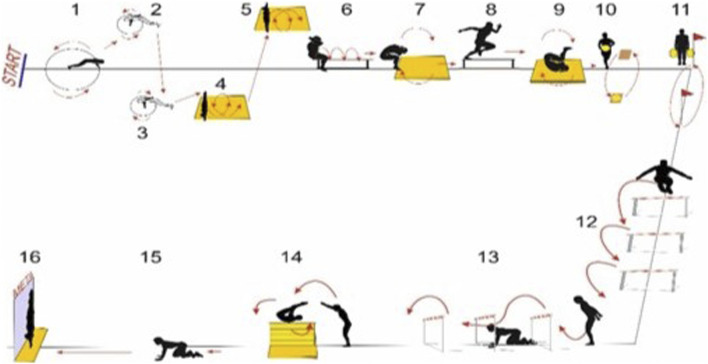
ASET diagram (Wochyński, 2015).

The Aviation-Synthetic Efficiency Test (ASET) is one of the most effective tests designed to diagnose the fitness of military pilots. It was developed by Wochyński and Stelęgowski in 2010 in Poland (in Dęblin) at the Air Force Academy in order to apply it to assess pilots’ ability to perform the anti-overload manoeuvre, and also to assess the fitness aptitude (motor adaptation) of pilots in their working environment (Wochyński et al., 2010). ASET has been consistently examined for a number of years. In 2015 it was modified by (Figure 1) Wochyński (2015). The test is used to evaluate the targeted training process. In recent time it has been used to evaluate special training on Special Air Gymnastic Instruments (Wochyński et al., 2021). ASET has been developed on the basis of current knowledge in physiology and aviation medicine. It consisted of 16 exercise stations set over a distance of 60 m. ASET training stations are designed to engage skeletal muscles that will be loaded during anti-G strain maneuvers tested in a centrifuge and flight missions. The negative correlation found between the achieved ASET execution time and the overall spin time on the overload centrifuge shows that the shorter the time achieved on the ASET the longer is the total spin time on the overload centrifuge (Wochyński et al., 2009). This fact confirms the usefulness of ASET in the pilot training process. ASET significantly correlates with 100 m, bar pull-ups and 1000 m, indicating a comprehensive nature of the effectiveness of motor ability assessment in pilots. It was observed that with pilots’ maximum performance (load) scores in covering the distance of 100 m, pull-ups on the bar and 1000 m run during the fitness test, the time to complete the ASET was prolonged (worse performance). ASET has been found to be extremely useful for pilots in terms of applying the physical load optimization criterion in the pilot training process (Wochyński et al., 2010; Wochyński, 2021). Based on many years of training experience, in addition to the examined correlation between ASET and 1000 m, 100 m and pull-ups on the bar, it appears that the amount of physical load in the above-mentioned sports (competitions) is of tremendous importance. Better ASET time executed by pilots means that the applied physical load is not exceeded in the process of motor preparation for flights (coordination optimization). Optimization of the training process load can be achieved by maintaining a statistically significant correlation between ASET and the analytical test consisting of 100 m, pull-ups on the bar and 1000 m. For this reason, it is necessary to use ASET and 100 m, pull-ups on the bar, 1000 m at the beginning and end of the training process in order to control the load. If necessary, the control of the training load can also be used in the middle of this process and the training emphasis can be corrected. Failure to demonstrate a statistically significant correlation, e.g.,: ASET and pull-ups on the bar, the trainee will not achieve the best result in overcoming ASET. ASET is a practical tool to accurately assess the optimization of physical load in the pilot’s flight preparation. It seems that the conducted analysis of the physical fitness of soldiers worldwide points to the use of tests for comprehensive motor assessment in their working environment. Such an analysis of soldiers’ physical fitness based on ASET is fully justified (Tomczak, 2024). A factor which supports such a focus of motor skills in pilots is the monitoring of changes in body components such as fat mass (FM), fat-free mass (FFM),muscle mass (MM), total water content (TBW), extracellularwater content (ECW), and intracellular water content (ICW), as well as in athletes in various sports disciplines (Mathunjwa et al., 2020; Witkowski et al., 2021; Chwałczyńska et al., 2021; Rutkowski et al., 2020). Recently, research has been published on optimizing the training process of cadet pilots taking into account body components (Prokopczyk and Wochyński, 2022) and also comparing athletes to cadet pilots in terms of the dynamics of body component changes under the influence of special programmes (Wochyński, 2022). The studies showed that physical fitness increased in the applied tests in terms of points in the group of judokas and pilots after the training process compared to the initial values. As a result of the low-intensity training process on SAGI and rapid fat burning, a significant correlation between ASET and FM was observed in cadet pilots. Higher training intensity and slower fat burning in judokas was confirmed by the insignificant low correlation between ASET and FM. The type of physical effort, intensity, body mass, changes in body components lead to the development of a tailored diet and appropriate hydration, which will increase the physical efficiency of pilots and athletes.

## Assessment of motor aptitude in terms of the pilot’s working environment

The motor predisposition of high-maneuvering pilots should be considered an essential condition for their selection for this type of profession (Wochyński et al., 2020). Solving this problem would bring tangible training benefits, cost savings and increased safety in the execution of air missions. This matter was a frequent subject of discussions, yet activity in this direction was absent due to the lack of convincing arguments. During the training process in the pilots’ physical preparation for flying, it could be seen that the cadets’ fitness levels varied. In this situation, the training objective was achieved merely by few participants in the allotted time. Other participants of the training process were either reaching the target with a long delay or faced other issues with the execution the exercises relevant for pilots. Determining motor aptitude for the pilot profession during their selection is a challenging task. It undoubtedly requires a great deal of experience and expertise in aviation physiology to apply an appropriate selection method. Opinions about the motor types of candidates who proved suitable for service in the Air Force were not uniform. There were opinions that the most important skills in a pilot are strength and speed. On the other hand, endurance plays the least important role (Wojtkowiak et al., 2006). Training observations showed that people with a predisposition to one motor skill, e.g., strength or speed, as well as endurance, did not show good results in extreme conditions of the pilot’s work environment. This was visible during overcoming the ASET, as well as in tests on the centrifuge. It was then decided to use an indirect test to determine motor types and compare them after overcoming the ASET. The determination of motor types and predispositions was made based on 40 m, 2000 m, pull-ups on the bar and ASET (Wochyński et al., 2020). This method of procedure resulted in faster achievement of the training goal. The recent research conducted in a centrifuge proved that the longest total spinning time, of interval characteristics was achieved by cadets with strength-endurance skills, and the weaker results were reached by a group of speed-strength skills. This study was additionally monitored by means of a lipid index, which confirmed this fact (Wochyński et al., 2016). The use of ASET was highly justifiable because of its comprehensive assessment of these characteristics, also determining the professional suitability of cadets for aviation. Due to the ASET diagnostic function within the motor adaptation in the pilot’s environment, the authors decided to determine the motor types using the Schele biopsy examination (Schele and Kaiser, 1982). ASET may have a more important selection role than before (Wochyński et al., 2010), because previously the motor types among cadets remained unspecified. The classification of motor types among cadets, and then ordering them to perform ASET gives the exact method of determining the motor capabilities for military aviation. Specifying the motor skills is the primary factor for the selection in aviation. Although we have a tool for evaluating maximum strain tolerance capabilities (centrifuge), there is no practical indirectly method of determining the pilot’s motor skills, which definitely hinders the choice of aviation candidates. Taking into account the groups of skeletal muscles involved in the anti-strain manoeuvre (manoeuvre L-1) during the test on a centrifuge and also during the ASET execution, the author’s own investigation confirms the fact that the endurance-strength type is suitable for the Air Force service, including body weight. In this case, a study was conducted to divide the candidates into two groups (A and B) according to their body weight (Wochyński et al., 2020). Respondents in group A were characterized by body weight of up to 73 kg and group B by body weight of more than 73 kg (Table 1). The division of groups was made based on the average body weight of all subjects.

**TABLE 1:** Speed (mean and SD) achieved during the ASET execution by all the examined cadets (n = 59), in group A (n = 30) and group B (n = 29) with a predisposition for endurance and strength (Wochyński et al., 2020).

Subject	En-St speed *(m ∙ s –1)*	St speed *(m ∙ s –1)*	En speed *(m ∙ s –1)*	En-StL speed *(m ∙ s –1)*
All	1.42 ± 0.10 (20)	1.37 ± 0.08 (14)	1.37 ± 0.12 (7)	1.32 ± 0.15** (18)
Group A	1.39 ± 0.12 (14)	1.29 ± 0.09 (7)	1.46 ± 0.10 (2)	1.35 ± 0.16 (7)
Group B	1.44 ± 0.07 (6)	1.44 ± 0.09 (7)	1.33 ± 0.05***† (5)	1.29 ± 0.15* (11)

En-St endurance-strength motor type; St strength motor type; En endurance motor type; En-StL endurance-strength motor type with low efficiency (lack of predisposition); ***the difference statistically significant in relation to the En- St at p < 0.01; **the difference statistically significant in relation to the En-St at p < 0.02; *the difference statistically significant in relation to the En-St at p < 0.05; †the difference statistically significant in relation to the St at p < 0.05; () number of examined individuals.

In the group of all the participants, the endurance-strength (En-St) motor type achieved the highest average speed over ASET than the endurance-speed type and the speed-strength type (Wochyński et al., 2020). In group A, the endurance/speed type achieved better average speed results over the ASET than the endurance-strength and speed-strength types (Wochyński et al., 2020). In group B, the speed-endurance type achieved a better average speed over ASET than the endurance/strength and endurance/speed types. This fact showed that the motor skills of the examined individuals, taking into account body weight in groups A and B, exert an effect on speed in the ASET execution. In group B, the candidates showed a dominance of endurance and strength motor skills with a statistically significant difference in height and body mass compared to group A. Research indicates that diagnosing motor abilities with body mass has a major impact on the effectiveness of pilot preparation for flights (Wochyński et al., 2020). Below, there is a practical example with a description.

**FIGURE 2:**
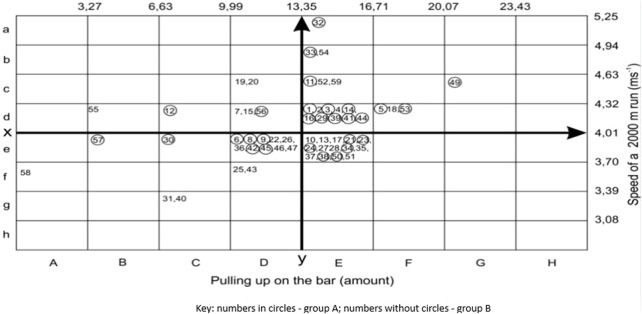
Results obtained in the strength and endurance test (Wochyński et al., 2020).

The x-axis in Figure 2 was average calculated from 59 examined cadets in pull-ups on the bar (13.35 amount ±3.36). The upper half of the table contained the results increased by one standard deviation (+1 s) (13.35 + 1.3.36 = 16.71), by 2 standard deviations (+2 s) (13.35 + 2.3.36 = 20.07) and by 3 standard deviations (+3 s) (13.35 + 3.3.36 = 23.43). The bottom half of the table contained results lower than the mean value, decreased by one standard deviation (−1s) (6.96−1.0.45 = 9.99), by 2 standard deviations (−2 s) (6.96−2 0.45 = 6.63) and by 3 standard deviations (−3 s) (6.96–3.0.45 = 3.27) (Wochyński et al., 2020). The y-axis ran vertically across exercise 2 and constituted the mean of the results of 59 cadets in the race over a distance of 2,000 m (4.01 m/s ± 0.31) by dividing it into the left and right side. The right half of the table contained the results higher than a mean value increased by one standard deviation (+s) (4.01 + 1.0.31 = 4.32) by 2 standard deviations (+2 s) (4.01 + 2.0.31 = 4.63) and by 3 standard deviations (+3 s) (4.01 + 3.0.31 = 4.94). The left half of the table contained the results lower than a mean value increased by one standard deviation (−1s) (4.01 + 1.0.31 = 3.70), by 2 standard deviations (−2 s) (4.01−2·0.31 = 3.39) and by. 3 standard deviations (−3 s) (4.01−3·0.31 = 3.08) (Wochyński et al., 2020). Figure 2 shows individual results of seven examined individuals with a predisposition to an endurance race. They showed a negative correlation with the results of strength attempts, including two with endurance predisposition above +1s. In fourteen examinees with a predisposition to strength, there was a negative correlation to the results of the endurance race. Twenty cadets showed aptitude for endurance and strength including four over +1 s, two over +2 s and one over +3 s for endurance running, while three over +2 s and one over +2 s for strength. Eighteen cadets showed a lack of predisposition to endurance and strength (Table 1). Two cadets in group A showed a predisposition to endurance running and five in group B. Seven subjects in group A showed a predisposition to strength and seven in group B. Fourteen cadets in group A showed a predisposition to endurance and strength and six in group B. Seven cadets from group A and eleven from group B showed a lack of predisposition to endurance and strength (Table 1). Among all the examined ones with a predisposition to endurance and strength, the highest speed of executing ASET was demonstrated by the En-St motor type, reaching an average speed V = 1.42 m/s. The author observed that all the participants performed the exercises on ASET at a speed which was statistically significantly higher (at p < 0.02) in case of the En-St motor type compared to the endurance-strength low performance motor type (En-StL). Group B also showed higher ASET overcoming speed in the En-St type compared to the endurance (En) and En-StL motor types, respectively, at p < 0.01 and p < 0.05. In group B, the strength motor type demonstrated a higher speed of performing ASET in relation to En at p < 0.05 (Table 1) (Wochyński et al., 2020). The study showed that a motor type with two motor skills, e.g., endurance and strength, is more useful than a single motor type, e.g., endurance (Table 1; Figure 2). As can be seen from Figure 2, the best endurance and strength result will be placed in box aH, and the worst in box hA. The best endurance result will be placed in box aA, and the best strength result in box hH (Wochyński et al., 2020).

## Assessment of vegetative-vestibular system habituation in pilots

One of many components of pilot physiological diagnosis is the assessment of habituation of the vegetative-vestibular system to the conditions of flight mission accomplishment. Undoubtedly, in military aviation, habituation of the vegetative-vestibular system in pilots is achieved by means of exercises on Special Aviation Gymnastic Instruments (SAGI), which include looping, gyroscope and the Rhine wheel. The general appearance of these devices was presented in figures in previous studies (Wochyński et al., 2010; Wochyński and Sobiech, 2014; Wochyński and Sobiech, 2015; Wochyński and Sbiech, 2017). Exercising on SAGI, by affecting the vestibular organ, can improve body stability. The basis for achieving vestibular organ habituation is the required adequate physical fitness of the exercisers prior to the start of the training programme on SAGI. Considering the importance of targeted strength exercises in improving the acceleration tolerance of +Gz pilots and the role of rotational exercises in shaping spatial orientation, a research experiment was conducted using training on SAGI. For training purposes, especially those concerning vestibular organ habituation (increasing resistance to airsickness), a detached loop test was used, verified in practical terms at the Polish Air Force Academy for several years (Jędrys, 1992). The detached loop test (Jędrys test), in contrast to the Coriolis test, differs in that it is performed over a longer period of time and in the standing position, and is specifically designed to assess the effects of the training process concerning vestibular organ habituation and spatial orientation. The test is of great diagnostic importance for the autonomic nervous system of pilots preparing for high-altitude flights. Vestibular organ habituation and spatial orientation are of great diagnostic importance for the autonomic nervous system of pilots preparing for high-altitude flights. Obtaining a statistically high time difference in the performance of this trial at p < 0.000001 on completion of the training programme, compared to the trial before the training programme demonstrates a successful application of the training programme on SAGI as well as the achievement of vestibular organ habituation and the reduction of adverse autonomic nervous system responses (Wochyński et al., 2020). This resulted in a significant improvement in angular acceleration tolerance and increased tolerance to motion sickness. The conducted research is the basis for using this method (Jędrys test) for special flight preparation exercises. It is noteworthy that vestibular habituation occurs faster and is remembered longer even under weaker stimuli. The vestibular habituation process is believed to involve not only peripheral receptors, but also subcortical centres of the brain, above all the reticular formation (Traczyk and Trzebski, 2007). The performance of this test does not require an additional device and is performed on a device (looping) used to train these features, which are essential during an air mission. The test on an unlocked looping is always accompanied by physical effort, which is absent in the rotational Coriolis test (the Bárány rotational chair test). The standing position, while performing this test, also gives information about an orthostatic capability, which undoubtedly plays a significant role in the pilot’s and astronaut’s organism. Under the influence of vertical positioning, the venous blood returns to the heart. The stimulation of the sympathetic system, in response to the irritation of the baroreceptor reflexes, leads to increased vascular resistance and arterial pressure. In healthy individuals, the baroreceptor reflex positively affects cerebral perfusion. The occurring postural hypotension is a symptom of an impaired function of the sympathetic system. As a result of the SAGI training process, the Jędrys test was performed, which has a long-term impact on the body, diagnosing the level of resistance to motion sickness and orthostatic abilities, which determines the safety of flights of cadet pilots. Postural hypotension causes cerebral hypoperfusion with such symptoms as loss of consciousness, dizziness, blurred vision, balance disorders associated with vertical positioning (Tachtsidis et al., 2005). After flights in outer space, it was possible to observe reduced orthostatic tolerance in a vertical positioning test, manifesting itself in a decline in the blood volume, increased blood retention in the lower limbs and a decrease in heart output (Buckey et al., 1996). It seems that, contrary to some opinions (Kłossowski, 1994), SAGI practice exerts a beneficial effect on the tolerance of accelerations + Gz. This fact is confirmed by the results achieved in overcoming ASET (Wochyński et al., 2021). Performing rotations on SAGI is a typical effect (workout) on the circulatory-respiratory system as well as low- and high-pressure baroreceptor reflexes caused by accelerations + Gz (head -feet direction) and - Gz (leg-feet direction).

The exercises on SAGI seem to have a tremendous importance in the early stages of flight training for cadet-pilots, preventing adverse factors which lead to space motion sickness and spatial disorientation. They also exert a beneficial effect on the level of general and targeted physical fitness (as measured by ASET) and the habituation of the vestibular organ. The detached looping test and its details were described in an earlier study (Wochyński et al., 2020). The attempt was interrupted immediately when the symptoms of space motion sickness occurred (Jędrys, 1992).

## Diagnostic value of lipid indexes in pilots

The use of the WS lipid index for pilots was suggested as early as 10 years ago (Wochyński et al., 2008). A was system of targeted fitness training for cadets, which requires checking its efficiency on a G-force centrifuge, by applying acceleration that reflects the characteristics of an air manoeuvre in combat. Wochyński and Sobiech’s lipid index was used for the overload centrifuge study to achieve confirmation of the physical capabilities of cadet pilots (Wochyński et al., 2016). The diagnostic function of the lipid index WS is that its value increases with increasing fitness, while the reverse trend means a decrease in the value of this index (Wochyński et al., 2001). ST muscle fibers are mainly involved in endurance exercise (Schele and Kaiser, 1982). The work of skeletal muscles involved in performing the overload maneuver could have an impact on the behavior of lipids in blood serum. The correlation between lipids and total centrifugation time would be of great diagnostic importance in terms of the pilot’s body performance during the test in the overload centrifuge. As lipids correlate with ST-type muscle fibres (Tikkanen et al., 1991), an attempt was made to evaluate the body load of cadets on an overload centrifuge with a stimulus that is a variant of the characteristics of interval-type accelerations.

The tests were administered to 19 cadets of the first year at the Air Force Academy, mostly 19 years of age, studying on the course of an aircraft pilot. The examined individuals were split into two groups, depending upon the level of obtaining the total time of centrifuging. Group I consisted of cadets who obtained the total time of centrifuging up to 254.2 s, during the first examination, whereas group II included cadets above this value. It was the highest value achieved in Group I.

The programme activities within the targeted training were conducted three times weekly, 2 h each, for 45 days which ended the half-year fitness preparation for flights. The exercises were designed to do general fitness exercises, paying particular attention to the work of muscles, being isometric in their character. The load was applied to those groups of muscles which were involved in the L-1 tension manoeuvre. On the basis of electromyographic testing they include m. latissimus dorsi, m. intercostalis, m. buccinator m. sternocleidomastoideus, diaphragma, m. pectoralis major (Chen et al., 2004) and m. flank femoris, m. flank at m. erector spinae (Oksa et al., 1996).

All the examined persons had identical accommodation conditions and diet. The test material was the blood taken from the basilic vein twice, before spinning and immediately after centrifuging (examination I) and also at the end of the 45 days period of training (examination II). The following were determined in the blood:

$$WS=\frac{\left(HDL+\frac{TG}{6}\right)\times \frac{Apo-A_{1}}{30}\times 10}{\left(LDL+\frac{TG}{5}\right)\times \frac{Apo-B}{20}}$$

where:

WS - lipid indicator of Wochyński and Sobiech

HDL - high density lipoproteine (mg/dL)

LDL - low density lipoproteine (mg/dL)

TG - triacylglycerols (mg/dL)

“6” -TG percentage share in HDL lipoproteine

“5” -TG percentage share in LDL lipoproteine

Apo-A_1_- apolipoproteine A_1_ (mg/dL)

Apo-B - apolipoproteine B (mg/dL)

“30” -Apo-A_1_ percentage share in HDL lipoproteine

“20” -Apo-B percentage share in LDL lipoproteine

10 - multiplier

The obtained findings from comparing the test groups at the beginning and at the end of the physical training period, before and after centrifuging, demonstrated that the shorter total centrifuging time, observed in group I involves a lower value of the lipid WS indicator, in contrast to group II, where an opposite direction of changes was recorded.

The lipid index WS, which positively correlated with the total spinning time in group II in the second study, before spinning (r = 0.62) and after spinning (r = 0.65) may be a useful diagnostic indicator of the function of skeletal muscles of the ST type.

The obtained correlations of the lipid index WS in groups I and II with the total spinning time highlight the greater strength-endurance of group II compared to group I.

The individuals from group I, above the value of WS lipid index equal to 6.80 and below the mean value of 262.74 s of the total centrifuging time are just resilient but not strong. In group II, participants with a lipid index WS above the average value of 10.06 and a total spinning time of 399.95 s were strong and enduring, while those below this value exhibited these traits at a slightly lower level than the average total spinning time.

Studies have shown that the lipid index WS had diagnostic value in differentiating the exercise capacity of cadets during centrifuge tests and in assessing their health status. Due to the diagnostic value of the lipid index WS, an analysis was conducted of the arguments supporting its use in aviation medicine as an additional important marker for assessing fitness and qualifying pilots for centrifuge tests (Wochyński et al., 2008). This analysis was confirmed by the author’s own studies, which demonstrated that cadets who achieved a higher total interval spinning time in the centrifuge had a higher lipid index WS during centrifuge testing. In the author’s own research it was also indicated that the indicator is of high diagnostic value, differentiating the groups: control, diet, exercise and diet, which is important from the point of view of monitoring the maximum physical performance and the diet of athletes and soldiers (Wochyński and Sobiech, 2004).

A reinforcing factor in the diagnosis of exercise and diet is Wochyński’s development of the VLDL-TG index, which along with the WS lipid index accurately interprets the body’s physical capacity and diagnoses disorders of fat metabolism and makes it possible to use dietary supplementation in athletes and in disease units. The VLDL-TG lipid index was presented in 2018 at the International Scientific Conference on Sports Medicine (Gdynia, Poland) (Wochyński and Majda, 2018):

$$TCh‐\left[HDL‐\left(\frac{TG}{6}+\frac{Apo‐A_{1}}{30}\right)+LDL‐\left(\frac{TG}{5}+\frac{Apo‐B}{20}\right)\right]=VLDL‐TG$$

where:

TCh - total cholesterol (mg/dL)

HDL-high density lipoproteine (mg/dL)

LDL - low density lipoproteine (mg/dL)

TG - triacylglycerols (mg/dL)

“6” -TG percentage share in HDL lipoproteine

“5” -TG percentage share in LDL lipoproteine

Apo-A_1_ - apolipoproteine A_1_ (mg/dL)

Apo-B - apolipoproteine B (mg/dL)

“30” -Apo-A_1_ percentage share in HDL lipoproteine

“20” -Apo-B percentage share in LDL lipoproteine

VLDL-TG - very low density lipoprotein triacylglycerols (mg/dL)

The above formula has high diagnostic applications together with the lipid index W S for the study of pilots in terms of optimal preparation for the L-1 overload manoeuvre.

## Diagnostics of the pilot’s psychomotor level

Diagnostics of the psychomotor level of pilots are among the most important determinants of pilot’s safety in flight operations. In modern special pilot training, it is essential to seek a psychomotor test adapted to the maneuverability of highly maneuverable aircraft. Assessing mental fitness in the extreme conditions of a pilot’s working environment is a novel feature. The assessment consisted of using a diagnostic and training device in the course of cognitive processes in conditions of dynamic change of body position in space (performing turns on a loop). The device enables the diagnosis of the level of functioning of the pilots’ mental reactions in extreme conditions of their work environment. The level of physical and mental efficiency depends on the course of the process of adaptation and integration of the two systems: the motor and the sensory one. In the timing criteria of executing the mentioned tasks, the level of physical and mental fitness may be the cause of the occurrence of feedback between these systems. During the application of the psychomotor test, a large discrepancy in the percentage of tasks performed and a smaller number of turns on the loop by the pilot were shown. Such results were caused by poor integration of the sensory-motor system. To this end, a new method of assessing psychomotor performance close to the real conditions of an air mission was used at the Air Force Academy (Poland) (Prokopczyk and Wochyński, 2022; Wochyński et al., 2023). A psychomotor test on a looping with a diagnostic and training device to assess mental conditioning (ability to concentrate and focus) and motor skills and abilities (accuracy of task performance) was first presented in 2015 at the 63rd International Congress of Aviation Space Medicine in Oxford, England.

### Description of the method for assessing pilot psychomotor performance

The evaluation of this method consists in determining the limit point of the maximum psychomotor abilities of cadets with decreasing correlation between the percentage of task completion and the performance of turns on the loop. The effects were visible when comparing the trials before the training period with the trial after the training period, showing the maximum psychomotor point with the lowest correlation (Prokopczyk and Wochyński, 2022; Wochyński et al., 2023).

The process of diagnosing hand-eye coordination using a specially designed device allowed for precise determination of the degree of task mastery under conditions of limited reaction time. The device to assess the level of sight-movement coordination of the pilot consists of special goggles, computer, a relay system and a specially developed application program of simulating spatial events. The programme included three tests with three levels of difficulty, with the ability to adjust the programmed time range for the appearance of stimuli and the registration of the participant’s response. Each test is composed of five tasks (incentives) which may be applied in a different order. The programme determined the reaction time of the task and the correct answers of a cadet. If the pilot exceeded the reaction time to a given stimulus or gave an incorrect answer, the computer recorded it as an error. At the end of the test, a report was printed with the test results and their statistical development under conditions in which the pilot performed the exercise, for example, on a looping, Rhein wheel or gyroscope (Special Aviation Gymnastic Instruments) in special goggles. During the pilot’s execution of exercises on the Special Aviation Gymnastic Instruments, stimuli (tasks) were sent from the computer wirelessly to the goggles screen. The tasks consist in solving simple arithmetic calculations, differentiation of figures and colour and of simulated spatial events. If the pilot commits an error in the test, he is unable to move on to the test of a higher degree of difficulty. The pilot is obliged to repeat the test. An evaluation of sight-movement coordination plays a significant role in aviation due to accelerations which may lead to sight-movement malfunction. A diagnostic-training device forces the pilot to activate thinking processes, increasing their powers of concentration and efficiency of sight-movement coordination (Prokopczyk and Wochyński, 2022; Wochyński et al., 2023). Table 2 presents the effects of the psychomotor skills level of cadets before the training period (test I) and after the training period (test II).

**TABLE 2:** Physiological and psychomotor parameters results in cadets (n = 20) (Wochyński et al., 2023).

Parameters	Test I M ± SD	95% CI	Test II M ± SD	95% CI	Cohen’s d test	F	p
Systolic pressure before the exercise [mmHg]	141.1 ± 15.2	133.9; 148.2	133.6 ± 13.11	127.5; 139.7	0.45	2.76	0.09
Diastolic pressure before the exercise [mmHg]	89.7 ± 9.91	85.1; 94.3	90.7 ± 10.16	85.9; 95.4	0.08	0.08	0.76
Systolic pressure after the exercise [mmHg]	154.9** ± 14.53	148.0; 161.7	143.7* ± 19.63	134.5; 152.9	0.55	4.16	<0.05
Diastolic pressure after the exercise [mmHg]	93.6 ± 15.37	86.4; 100.7	91.7 ± 11.75	86.1; 97.2	0.10	0.19	0.66
Heart rate (HR) before the exercise [bpm]	84.8 ± 15.96	77.3; 92.3	84.0 ± 13.27	77.8; 90.2	0.04	0.03	0.86
Heart rate (HR) after the exercise [bpm]	125.25** ± 24.38	113.8; 136.6	123.65** ± 17.92	115.2; 132.0	0.05	0.05	0.81
Execution of rotations forward on looping [number]	49.45 ± 10.5	44.5; 54.3	54.1 ± 11.74	48.6; 59.5	0.34	1.74	0.19
Percentage completion of tasks during doing exercises on a looping [%]	73.7 ± 14.84	66.8; 80.6	87.25 ± 10.85	82.1; 92.3	0.81	10.77	<0.01

M±SD-mean, standard deviation; CI- confidence interval; p-level of significance *p < 0.05 - statistically significant difference in comparison to the pre-exercise value; **p < 0.001 - statistically significant difference in comparison to the pre-exercise value.

Application of the newly developed evaluation system of sight–movement reactions for pilot’s examination, combined with an analysis of the results of the conducted biochemical, physiological and psychomotoric examinations facilitated to extend the diagnostic value of the issue and find replies to the following questions: 1. How does the pilot function in conditions of angular accelerations, with a simulation of different flight characteristics on the SAGI? 2. How many errors does he commit? 3. Is he capable of concentrating well and efficiently carrying out the tasks he faces? 4. What is the psychophysical condition of his whole organism? (Wochyński et al., 2010).

## Biochemical and physiological indicators in monitoring the special training process in pilots

From the point of view of flight safety, monitoring a pilot’s fitness in an extreme training environment is an important factor in conducting a combat aviation mission. The exploited exercises on the Special Aviation Gymnastic Instruments (SAGI) aim at preparing the pilot’s organism for extreme conditions in the working environment. The available research demonstrates that exercises on SAGI improve psychomotoric efficiency (Kobos et al., 1994; Kobos et al., 2013). Monitoring adaptation to optimal pilot working conditions through biochemical indicators is a novel approach. Therefore, the author attempted to find sensitive biochemical markers in order to evaluate adaptation and post-exercise body load. Due to high physical and mental body overloading in the exercising conditions on SAGI, the study takes into account metallothionein (MT), zinc (Zn), copper (Cu), protein and neuron-specific enolase (NSE) in the blood serum (Wochyński and Sobiech, 2014). Due to the diagnostic function of proteins and enzymes in urine, depending on the degree of the induced metabolic reactions during a physical exercise, the authors used ß-2 microglobuline (ß-2M)), albumin (ALB), total protein (TP), N-acetyl-β-D-glucosamine EC. 3.2.1.30 (NAG) expressed in creatinine (Cr) as post-exercise markers. Studies have been undertaken in the training process of cadets on SAGI on serum levels of Apo-E and Lp-(a) (Wochyński et al., 2011). The study attempted to assess the diagnostic suitability of the above proteins in order to monitor the training load of cadets on the SAGI, compared to the control group, whose physical effort and intensity were of a different nature. At the same time on the basis of these exercises, the authors evaluated the general physical fitness in cadets as the consolidated total of the specific exercises on the SAGI.

The examination in question was conducted in 55 cadet officers (males), 20 years of age, who were then split into two groups: group A, which underwent testing (N-41), and group B, the control one (N-14) that followed the standard training physical education programme. In both groups, the material used for research was blood taken twice, before (BT) and after (AT) the workout; at the beginning (training I), in progress (training II), and at the end (training (III). The training cycle lasted 70 days. In both groups, the physical training period consisted of 22-h training units. In order to assess the intensity of the training, in both groups, POLAR TEAM-2 PRO system was used. It registered the changes in the heart rate (HR). The cadets were provided with identical food and accommodation conditions during the whole of the training period. The physical fitness of the cadets in group A and B was assessed at the beginning (series I) and at the end (series II) of the conducted investigation by means of the following fitness tests: Aviation-Synthetic Efficiency Test (ASET) (Wochyński et al., 2010), a shuttle running test 10 m × 10 m, pull-ups on a bar, 16.5 m race and forward bends. The studies covered the same groups of cadets, applying an identical procedure of conducting the investigation, measurement of intensity and physical fitness; the research material was blood and urine (proteinuria). The findings of the conducted investigation proved that the post-exercise load in cadets in group A and B, in the majority of the indicators, caused significant changes in AT. The difference in the changes between group A and B is due to the intensity of the physical effort, which was confirmed by the HR measurement. The lower intensity in group A is related to a shorter exercise time caused by longer intervals between changing the exercise instruments, and also by the nature of the physical effort. It seems that in group A, the exercises on the SAGI seriously burden the body system despite decreased intensity due to the occurrence of positive G-strain + Gz (head-feet direction) and negative G-strain -Gz (direction feet-head).

### Metallothionein function in physical exercise in cadet pilots

Previous studies showed that MT has a diagnostic value in soldier athletes at distances of 2,000 m and 4,000 m (Milnerowicz et al., 2004), in endurance-speed training and in the marathon (Wochyński et al., 2003). MT has been shown to be a good marker of physical exercise (Wochyński et al., 2003). Due to the MT structure and the specificity of physical exercise in pilots, it was applied to monitor the special training process. The obtained results of the examination (Wochyński and Sobiech, 2014) showed that the examined groups of cadet pilots varied in the character and intensity of the physical effort. The evidence was the difference in the concentration of MT, Zn, Cu, NSE and protein between group A and B. This was caused by lower intensity and nature of the load in group A, in comparison with Group B (control one). The nature of the physical effort on the SAGI showed a greater demand of the organism for protein, Zn and Cu. The lower concentration of MT in training III of group A, in comparison with group B, possibly results from the fact that the adopted training heavily overburdened the central nervous system and internal organs of the cadets, which brought about better results obtained on the ASET, and which, among others, determines movement coordination. In this case, metallothionein plays the role of an antioxidant (regulator) by combining its function with the metabolism of protein, Zn and Cu. MT is an effective marker in guiding the training process. Due to the fact that MT is an acute-phase protein and a scavenger of free radicals generated during exercise (Milnerowicz et al., 2004), further studies on this protein should confirm its diagnostic significance.

### Post-exercise proteinuria in cadet pilots

The proteins secreted into the urine can provide valuable information about the process of exercise adaptation. Post-exercise proteinuria occurs directly after physical effort and may continue even 24 h after its completion. The key role in the formation of stress proteinuria is exercised by kidneys (Ghieda et al., 2011; Bakońska-Pacoń and Borkowski., 2003). The cause of the increase in the concentration of protein in conditions of intense physical effort is the penetration of the plasma proteins to renal tubules (Poortmans, 1985). An increase in proteinuria depends upon the degree of adaptation to exercise zone of aerobic and anaerobic metabolic changes (Poortmans and Labilloy, 1988). Increased proteinuria was found in the zone of anaerobic changes, rather than in aerobic ones. It was also observed that individuals who were not well-prepared showed a greater increase in protein levels in their urine.

Comparing the pre- and post-training values of the protein indexes normalized to creatinine in training sessions I, II, and III between both groups, it should be noted that group B exhibited higher proteinuria compared to group A, alongside a higher intensity of physical exertion (Wochyński and Sobiech, 2015). The observed correlations in group A indicate short duration filtration barriers on the level of the damaging to the resorptive function of the renal tubules, as proved by the positive correlation between NAG/Cr and ß-2M/Cr. In Group B, however, the negative correlation between NAG/Cr and ß-2M/Cr may indicate an interaction of these two mechanisms together (Wochyński and Sobiech, 2015).

The results of the comparison between groups A and B showed that the AT values of TP/Cr index and NAG/Cr index differentiate the intensity of physical activity, and are good markers of effort adaptation. The observed correlation between the NAG/Cr index and the ß-2M/Cr index in groups A and B provides a basis for diagnosing the type of physical exertion and the type of proteinuria. In group A, the positive correlation observed at the end of the training period (training III) between the TP/Cr index and the ß-2M/Cr index, as well as between the NAG/Cr index and the TP/Cr index, may serve as an indicator for assessing the degree of kidney strain after training and potential short-term damage to the glomeruli and renal tubules. It was observed that the type of proteinuria has an impact on the achievement of exercise adaptation in a specific area of metabolic changes.

### Post-exercise concentrations of apolipoprotein E and lipoprotein -(a) in cadet pilots

Changes in apolipoprotein E (Apo-E) and lipoprotein-(a) (Lp-a) levels in serum, after exercises on the SAGI, give a practical possibility to diagnose the functioning of certain internal organs and the status of blood vessel walls in the process of post-exercise adaptation, which from the standpoint of a pilot’s preparation for conducting tasks in extreme conditions is undoubtedly of tremendous importance (Wochyński et al., 2011).

The obtained findings demonstrated that the concentration of Apo-E and Lp(a) in trainings I, II and III immediately after the physical effort falls in both groups. It should be stressed that the applied exercise did not induce changes which could indicate inadequate load to the capabilities of the students. The examinations showed a significant difference in the values of Apo-E between the two tested groups. Higher values of Apo-E concentration level were found in Group A, in comparison with group B, which may be related to the nature or intensity of the physical effort. One should note a post-exercise drop in the concentration of Apo-E as opposed to the prior-exercise values, in both groups, in the three trainings, although it was only significant for Group A. This may result from a different load of internal organs or possibly their adaptation to the prescribed physical effort as well as the Apo-E genotyping domination. Apolipoprotein-E is synthesized in the liver, brain, spleen, kidney, lungs, muscles and in high concentrations in the interstitial fluid, involved in the transport of cholesterol from the cells with excessive cholesterol to those individuals who are in need of cholesterol (Mahley, 1988). Surprisingly, the Apo-E genotype has been shown to increase aerobic capacity stimulated by physical training, probably through an unspecified pathway on neural and skeletal muscle function (Thompson et al., 2004). The observed Apo-E rich in the VLDL cholesterol fraction is presumably a metabolically active particle fraction, whose level may regulate the level of triglycerides in plasma after physical exercises (Klein et al., 1992). In this study, a post-exercise reduction in the concentration of Apo-E in both groups A and B was caused (over a period of 70 training days) by the regularity and character of the physical effort and probably the genotypic composition. The obtained findings demonstrated that there is no significant difference between the prior-exercise and post-exercise values, in trainings I, II and III, in the concentration of Apo-E and Lp (a); a downward trend in the values of these parameters, from training I to training III, which undoubtedly results from the applied exercise programme. It is possible to achieve significant differences between the workouts, however they would need longer exposure times of the exercises on the body, accompanied by a modified diet. With regard to the physical fitness of the subjects, it can be stated that improvements in physical fitness are accompanied by a decrease in Apo-E and Lp(a) concentration levels in both study groups.

The significant difference observed between post-exercise and pre-exercise Apo-E concentration values in training I, II, and III in group A, compared to group B, may be attributed to the exercise load on the SAGI, where the predominance of concentric muscle work induces a short-term episode of significant reduction in Apo-E concentration. The results of these studies may be useful in enhancing physical performance through dietary changes and modifications to the training programme.

The applied physical fitness tests, along with observations of exercise markers before and after the training process (on SAGI) in group A, confirm exercise adaptation, showing better physical fitness compared to group B (the control group).

## Conclusion

In a modern pilot training system, monitored training is an essential element of flight safety. Failure to match physical fitness with the ability to perform the task may lead to an accident. Most frequently, accidents resulted from human error during the performance of aviation tasks. From a psychological point of view, they are the result of an imbalance between the demands of the situation and a person’s capabilities. Therefore, special attention has been paid to the effectiveness of fitness and physiological diagnostics for military pilots in order to prevent the impairment of human capabilities in extreme situations. The fitness diagnostics and practical tests applied above undoubtedly contribute to flight safety. Pilots should be a group that is aware of the permanent monitoring of physical activity and examined with the available tools. A pilot’s experience cannot be shaped solely by the number of flying hours without adhering to the principles of preparing the body for flights. The physical preparation process of pilots has a positive impact on the pilot’s psyche, fitness and their health, as well as enhancing their professional experience. A pilot who disregards such a preparation has less judgement in performing a task under extreme conditions and increases the risk of an aircraft accident. The cited research description should be the basis for further research in this area in the future to achieve habituation of the body under extreme exercise conditions as well as modifying the training methodology and developing an appropriate diet for pilots. The author of this work aimed to emphasize the great substantive and practical significance, as well as the innovative aspects in fitness, psychomotor, and physiological diagnostics, effectively enhancing flight safety.
